# Evaluation of Switch and Continuous Navigation Paradigms to Command a Brain-Controlled Wheelchair

**DOI:** 10.3389/fnins.2018.00438

**Published:** 2018-06-28

**Authors:** Álvaro Fernández-Rodríguez, Francisco Velasco-Álvarez, Manon Bonnet-Save, Ricardo Ron-Angevin

**Affiliations:** ^1^Departmento de Tecnología Electrónica, Universidad de Málaga, Malaga, Spain; ^2^IMS UMR Centre National de la Recherche Scientifique 5218, Cognitique et Ingénierie Humaine, Bordeaux INP-ENSC, Bordeaux, France

**Keywords:** brain-computer interface (BCI), wheelchair, navigation control, switch, continuous, usability

## Abstract

A brain-computer interface (BCI) is a technology allowing patients with severe motor dysfunctions to use their electroencephalographic signals to create a communication channel to control devices. The objective of this paper is to study the feasibility of continuous and switch control modes for a brain-controlled wheelchair (BCW) using sensorimotor rhythms (SMR) modulated through a right-hand motor imagery task. Previous studies, which used a continuous navigation control with SMR, have reported the difficulty of maintaining the motor imagery task for a long time, especially for the forward command. The switch control has been presented as a proposal that may help to solve this issue since this task is only used temporary for either disabling or enabling the movement. Regarding the methodology, 10 of 15 able-bodied users, who had overcome the criterion of 30% error rate in the calibration phase, controlled the BCW using both paradigms. The navigation tasks consisted of a straight path divided in five sections: in three of them the users had to move forward, and in the other two the users had to maintain their position. To assess user performance in the device management, a usability approach was adopted, measuring the factors of effectiveness, efficiency, and satisfaction. Then, variables related to the time employed and commands selected by the user or parameters related to the confusion matrix were applied. In addition, the scores in NASA-TLX and two *ad hoc* questionnaires were considered to discuss the user experience controlling the wheelchair. Despite the results showed that the best system for a specific user relies on his/her abilities and preferences, the switch control mode obtained better accuracy (0.59 ± 0.17 for continuous and 0.72 ± 0.05 for switch). Furthermore, the switch paradigm can be recommended for the advance sections as with it users could complete the advance sections in less time (42.2 ± 28.7 s for continuous and 15.47 ± 3.43 s for switch), while the continuous mode seems to be better at keeping the wheelchair stopped (42.45 ± 16.01 s for continuous and 24.35 ± 10.94 s for switch).

## Introduction

Diseases such as amyotrophic lateral sclerosis (ALS) or brainstem lesions may result in a deterioration of the motor functions of affected patients, who could need to use assistive technology to facilitate tasks in their daily lives. However, some patients could not be benefitted from conventional systems, such as joystick or eye-tracker systems, due to the severe reduction of their motor functions. Therefore, the solution could be systems that do not require the motor capacity of the users to control them. Brain-computer interface (BCI) fullfils this requirement since it is a technology that allows the use of electroencephalographic (EEG) signals to create a communication channel between users and the device that they want to control. These systems have been implemented in devices such as a speller matrix (Farwell and Donchin, 1988), a home automation system (Corralejo et al., 2011) or a wheelchair (Millán et al., 2009). The study of the control of a wheelchair through EEG, i.e., a brain-controlled wheelchair (BCW) is the objective of the present work. Since the first BCW-related publication in 2005 by Tanaka et al. (2005), numerous proposals can be classified considering different aspects of the system. The main taxonomies divide these wheelchairs depending on the EEG signals registered or the navigation system implemented (Fernández-Rodríguez et al., 2016).

Firstly, the EEG signal most used for the control of a BCW in real environments has been the sensorimotor rhythms (SMRs) (e.g., Millán et al., 2009). This endogenous signal is based on the event-related (de)synchronization (ERD/ERS) phenomenon: mu (7–13 Hz) and beta (13–35 Hz) bands amplitude variations in the sensorimotor cortex area while performing a motor imagery (MI) task. Therefore, the SMR can be freely modulated by users and applied as a control command in a BCW without needing external visual, tactile or auditory stimuli (Pfurtscheller et al., 2006). As a result, a SMR-based BCW could allow sensorial channels to be dedicated to the maintenance of attention to the environment, an important factor when controlling a wheelchair. This is an advantage vs. other BCW based on an exogenous signal, such as the P300 (e.g., Iturrate et al., 2009 or Zhang et al., 2016) or steady-state evoked potentials (e.g., Ng et al., 2015 or Kim and Lee, 2017, who used visual and somatosensory signal, respectively), which usually require a graphic user interface (GUI) for its control.

Secondly, the main taxonomy of navigation systems distinguishes low and high level categories. On the one hand, in low-level navigation systems, wheelchair control is achieved through simple navigation commands such as “move forward” or “turn right.” In this way, users can have a fine control and perform any path they want. On the other hand, high-level navigation lets users have a rough control of the BCW, selecting destination commands such as “take me to the kitchen” or “leave this room.” Although the high-level navigation might induce a smaller workload, since the user simply selects the destinations, the present study is framed within the low-level systems because they could be more appropriate for uncontrolled environments. In particular, low-level navigation should allow the desired flexibility to avoid obstacles or adapt the trajectory of the wheelchair if new modifications occur in the environment. This navigation could help to maintain an adequate engagement and improve the user's experience, since he/she has a main role controlling the wheelchair and a strongest feeling of autonomy. Likewise, there are two main types of low-level systems for controlling a BCW: discrete and continuous control. In discrete control, the selection of a navigation command implies a prefixed action, e.g., a turn of 45° or a fixed advance distance of 1 m (e.g., Tsui et al., 2011 and Ron-Angevin et al., 2017). Otherwise, in the continuous control the user can control the extension of the movement after the selection of a navigation command, e.g., the turn amplitude or the advance distance (e.g., Millán et al., 2009 and Li J. et al., 2013). Usually, in this last control the movement continues as long as the user keeps the command active.

Another paradigm was proposed by Mason and Birch (2000), Müller-Putz et al. (2010), and Solis-Escalante et al. (2010): the brain switch. Usually, the aim of this paradigm applied in asynchronous BCWs has been to offer an on/off device control (Xu et al., 2012; Cao et al., 2014). However, the brain switch concept can be also applied directly on the control commands of a BCW. That is, not only to turn on/off the system but, for example, to activate/deactivate the wheelchair's forward command. Following this idea, a hybrid exogenous (SSVEP and P300) based BCW using a similar interpretation to the brain switch control applied in the control command was presented by Li Y. et al (2013).

Nevertheless, the application of the brain switch paradigm in the control commands of a MI based BCW could offer a remarkable improvement. Thanks to the brain switch the user could be able to maintain a state, e.g., the advance command, without using the MI task for a long time. The switch paradigm has been previously used in a MI based virtual wheelchair by Velasco-Álvarez et al. (2010) and Huang et al. (2012). Besides the paper of Velasco-Álvarez et al. (2010) the BCI group of the University of Malaga (UMA-BCI) has applied this paradigm on the management of a real mobile robot using SMR (Ron-Angevin et al., 2015).

The switch paradigm adapted by the UMA-BCI group to control a BCW is used for the selection of the forward navigation command without needing to maintain the MI task during the displacement. Specifically, if the user wants to select a forward (when he/she has stopped) or stop (when he/she is moving) command, he/she has to perform the MI task. Otherwise, in order to keep the current state of the wheelchair (i.e., to continue the advance or the stop), the user has to carry out an alternative task (e.g., arithmetic operation). Therefore, the main point of the switch handling is that the MI task is only used to change the movement state of the wheelchair, not to maintain it. Moreover, this management allows the user, as in continuous mode, to control the exact distance of displacement. In continuous mode, the user must maintain the desired task stably: on the one hand, a task to select an active command (i.e., move forward) and, on the other hand, a task to remain immobile. However, in switch mode, participants must have the ability to perform one task quickly (related to changing the present state of the wheelchair), but should have a stable control of the other (related to maintaining the present state of the wheelchair). Although in the present work only one active command (besides the stop command) is used, the forward command, the obtained conclusions could be transferred to other paradigms with a larger number of commands. In addition, the simplicity of the design allows to isolate the object of study (i.e., the advance command in two control modes) and to establish a more reliable comparison. The detailed functioning of these paradigms will be presented in section Navigation Application.

Continuous concentration on a mental task for controlling BCI devices could be a tiring task that not all users can manage. This could be a considerable problem during the control of continuous navigation, for which at least two tasks must be stably controlled. Therefore, either because of the user's skills or the complexity of the task, sometimes it is difficult to find two tasks in which the user can maintain an acceptable performance over a long period of time. The switch mode could be a solution in which only one of these tasks should be maintained in a prolonged way: the task for keeping the current state. Due to the previously exposed, the switch navigation might improve the time needed to complete a path and the effort that the user has to employ carrying out the task.

In short, the brain switch paradigm could be a suitable option for controlling a real MI based BCW, especially for the forward command. Therefore, the present work will be focused on testing this hypothesis by comparing two navigation methods for a real wheelchair control: continuous and switch paradigms.

The approach used to study these paradigms will be based on the definition of usability given by the International Organization for Standardization (1998). According to them, this construct is divided into three factors: effectiveness, efficiency and satisfaction. For effectiveness, the user performance in controlling the BCW will be studied. The efficiency factor will take into account the resources used and costs to achieve the yield obtained. Finally, satisfaction will focus on measuring the user experience regarding comfort and subjective opinions about how they experienced controlling the wheelchair.

## Methods

### Participants

Fifteen able-bodied participants took part in the study (mean age 23 ± 3.44 years; 7 men, 8 women), identified as P1–P15 here. Most of them were students from the University of Malaga and only P4 had previous experience in BCI systems, but none in a BCW control. They were mainly recruited through the use of social networks and word of mouth, having been offered an economic reward for their participation. The study was approved by the Experimental Ethics Committee of the University of Malaga and met the ethical standards of the Helsinki Declaration. Participants stated that they had no medical history of neurological or psychiatric disorders in the written informed consent, nor did they take any medication regularly. All these subjects participated in an initial calibration task consisting in a first test examining the ability of subjects to control their SMR signal (see section Calibration Task). This study needed users to have acceptable control of their SMRs, which would enable them to control the BCW in the navigation task (see section Navigation Task With the Brain-Controlled Wheelchair). For this reason, as a design criterion in the calibration task, a conventional limit of 30% in the classification error rate was considered to be the maximum that could allow efficient control of the paradigm; the same limit was used in Kübler et al. (2001) for efficient communication using a two-class BCI for spelling. In a similar way, this study needed users to have acceptable control of their SMRs, which would enable them to control the BCW in the navigation task (see section Navigation Task With the Brain-Controlled Wheelchair). In the case of a classification error rate over 30%, participants were rewarded (5 €) and the experiment ended; otherwise, they continued to the real BCW control (10 €, regardless of their performance controlling the wheelchair).

### Data acquisition and signal processing

EEG signals were recorded at a 200 Hz sampling rate using the following electrode positions: F3, F4, C3, C4, P3, P4, T7, T8, and Cz according to the 10/20 international system. Ground and reference were placed at AFz and Fz positions respectively. Signals were amplified by an actiCHamp amplifier (Brain Products GmbH, Munich, Germany). These electrode positions were combined to generate two large Laplacian channels (for extended details see McFarland et al., 1997) over C4 and C3 which correspond to the right and left sensorimotor areas, respectively. Neither online nor offline artifact detection techniques were employed.

As mentioned above, users participated in an initial training session for calibration purposes. This exercise consisted in performing two mental tasks (80 trials for each task) during which the EEG signals of the users were recorded. These data were used to obtain a reactive frequency band and the classification error rate for each subject (detailed below) by an automatic process. The selected subjects were those with a classification error rate under 30% and their calibration parameters were obtained to be used during the control navigation task. Data processing and feedback generation in the navigation exercise were based on the procedure detailed in Ron-Angevin and Díaz-Estrella (2009):

- Although in some cases it is possible to find subjects whose reactive band belongs to the β band, the search for the optimal frequency band was limited to the μ band for simplicity. The reactive frequency band of each participant was automatically selected from all possible frequency intervals between 5 and 17 Hz (with a minimum bandwidth of 2 Hz). For each tested frequency interval, feature extraction, and classification were carried out, giving a frequency band-dependent error rate as a result. The band that led to the lowest classification error rate was regarded as the subject's reactive frequency band.
- Feature extraction: the average power of the signal from the two EEG channels (right and left sensorimotor areas) was estimated in the specific frequency interval for each trial. This average was calculated by (i) digitally band-pass filtering the EEG using a fifth-order Butterworth filter, (ii) squaring each sample, and (iii) averaging over several consecutive past samples. A total of 100 samples were averaged, giving an estimation of the band power for intervals of 500 ms.
- Classification: the error rate time course of a linear discriminant analysis (LDA) classifier (Lange et al., 1997) was computed using features from both channels by means of a ten-times ten-fold cross-validation scheme. In this way, the estimated minimum error rate of the classifier from the given frequency band was obtained.
- Feedback generation: the previously selected frequency band and the obtained parameters were used to set up LDA whose classification results determined the feedback “L,” which was used in the next sessions. This feedback was computed online every 31.25 ms. All data processing was carried out in MATLAB.

### Navigation application

In the present work two control paradigms have been studied: continuous and switch mode. However, the criterion to detect the mental tasks remained similar. Two mental tasks were used: an active task which was a right-hand motor MI, and an alternative task used as a distractor to prevent thinking about the right hand task (detailed in section Calibration Task). Performing the MI task was used to control the extension of a bar—called “L,” not visible to the user since the interface was only acoustic—as a result of the LDA classification. Specifically, if the classifier determined that the task performed was right-hand MI, the bar was extended; in other cases, its length remained at its minimum size. When the bar exceeded a selection threshold during a time larger than a “selection time” (around 1 s), a command selection was executed (the selected command depended on the paradigm handled). Besides, if the bar length was lower than the selection threshold for a period less than a “reset time,” the accumulated “selection time” was not reset, but otherwise it was set to zero. Both control modes started in a rest state (not possible to manage the BCW) from which the users have to activate the availability of the two control commands to begin the movement with the wheelchair. To change from the rest state to the control state, after hearing the word “wait” in Spanish, the MI task needed to be executed. As the user executed this, the word “advance” was played to indicate the availability of the forward command and the possibility to start to move. At this point, the control mode used conditioned the next event.

In the continuous mode, the MI task was destined only to move the BCW, i.e., when the user performed this task, it extended the abovementioned bar (“L”) and the device advanced continuously as long as the bar was over the selection time and threshold. Otherwise, to select the stop command, the user must perform the alternative task.

Regarding the switch mode, its control was similar to that employed for a light switch. If the user wanted to start an advance or to stop the wheelchair, i.e., to change the state of the wheelchair, he/she had to perform the MI task. On the contrary, if the user wanted to maintain the forward or stop command, he/she had to perform the alternative task.

**Figure 1:**
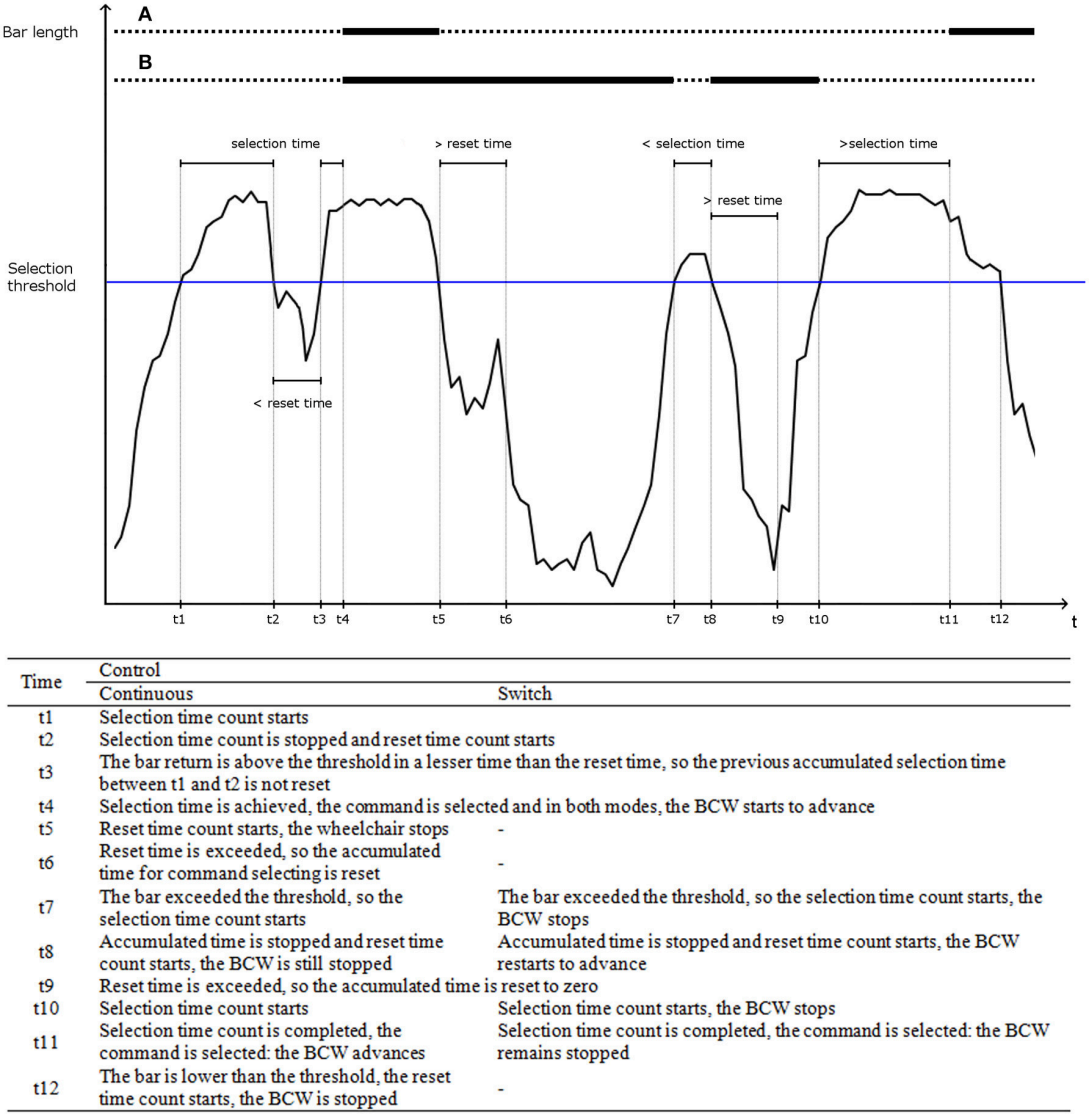
Operation of the interface through a detailed example. This example represents the bar length (axis *y*) over time (axis *x*) on the control of a brain-controlled wheelchair (BCW). At the beginning, the BCW is stopped. The horizontal lines *a* and *b* represent the executed command for the wheelchair for continuous and switch mode, respectively: a solid line for the forward command and a dashed line for the stop command. A detailed explanation of the events for continuous and switch control modes is offered at the bottom of the figure.

An illustrative example of the movement of the bar and command selections is shown in Figure 1.

### Robotic wheelchair

**Figure 2:**
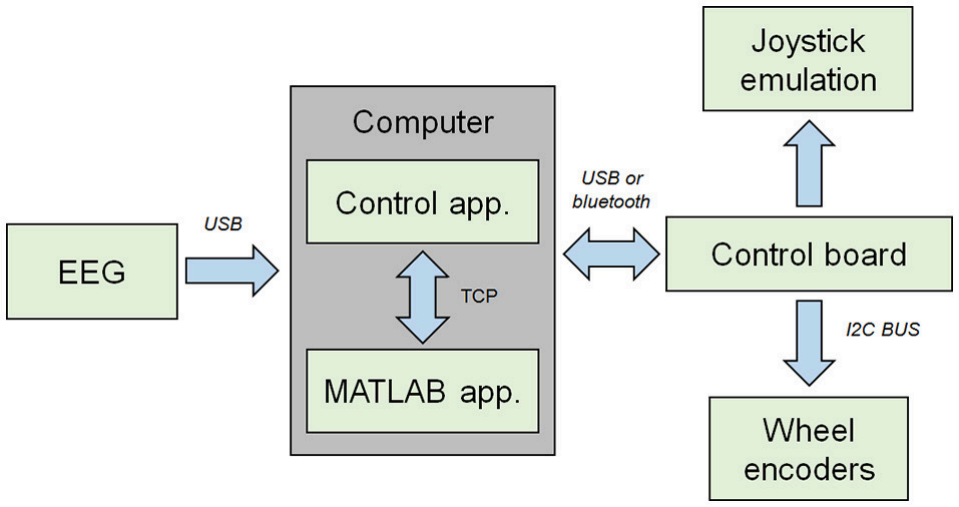
Module structure of the developed brain-controlled wheelchair.

The BCW used consisted of a customized Invacare Mistral3 electric wheelchair (Figure 2) equipped with a custom-built control board emulating its analog two-axis joystick in real time and receiving multiple sensor information through an I2C bus. This board was connected through a USB port to a control application written in C that ran on an external laptop. This application received, via a TCP connection, the commands (e.g., move forward) issued by the navigation application running in a MATLAB session, and then transformed them in real time into low-level commands that were fed back to the control board. Two AS5048 magnetic rotary encoders were attached to the wheelchair's driving wheels in order to carry out the odometry and thus compute the wheelchair's heading at every moment. This information was used by the application control to correct small drifts both online and just after having performed a displacement. The BCW took around 5–6 s to make a 1-meter advance.

### Procedure

**Figure 3:**
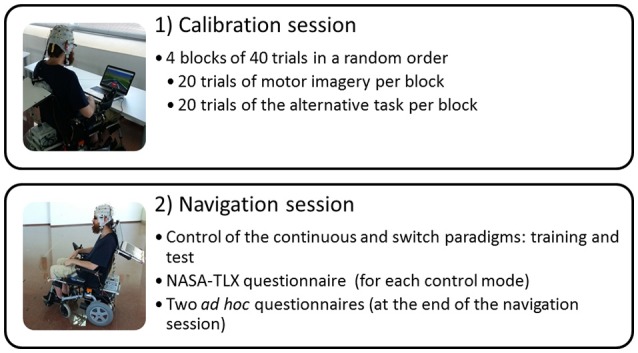
Experimental procedure.

The study consisted in two sessions per participant (Figure 3) carried out in 1 day with a total duration of approximately 2 h: (i) a calibration session to know the initial skill of users to control their SMR and to obtain their parameters, and (ii) a navigation session with the BCW to assess the feasibility of the paradigms through their execution and three questionnaires (presented below). Both the calibration and the navigation were performed in a quiet and spacious room of the Higher Technical School of Telecommunication Engineering of the University of Malaga. Prior to their session, users were informed via email about the task and the proceedings of the experiment. However, the relevant details were re-explained at the beginning of the session before signing the consent. All this preparation process, including the EEG montage, had an approximate duration of 20–25 min.

#### Calibration session

The present calibration session was based in the one previously used by Velasco-Álvarez et al. (2013) and consisted in a virtual environment guiding the user to perform two different mental tasks without any feedback. Hence, the aim of this phase was for the system to learn to recognize both user tasks when used as control commands. The user tasks were right hand MI and an alternative mental task (word chain or mental arithmetic) and were freely chosen by users who received some advice. Regarding the motor imagery task, they were advised to employ a fine MI-related fingers movement, using visual, and kinaesthetic imagery, while for the alternative mental tasks, on the one hand, the mental arithmetic should be difficult enough to maintain the user's focus but not to provoke frustration (e.g., to do a series of subtractions of 13 units starting from a random number between 90 and 300). On the other hand, the word chain task consisted of picking up some random word in Spanish and choosing another word whose first syllable was the same as the last syllable of the previous word (e.g., “*fies-ta*,” “*ta-pa*,” and “*pa-e-lla*”). If they were stuck with some word, they should pick another word, as the main objective of this task was just to remain concentrated on it. In addition, they were instructed to always use the same two specific tasks, to continue to watch the screen, to avoid any muscular movement, to try to reduce blinks and to maintain a relaxed and motivated state.

**Figure 4:**
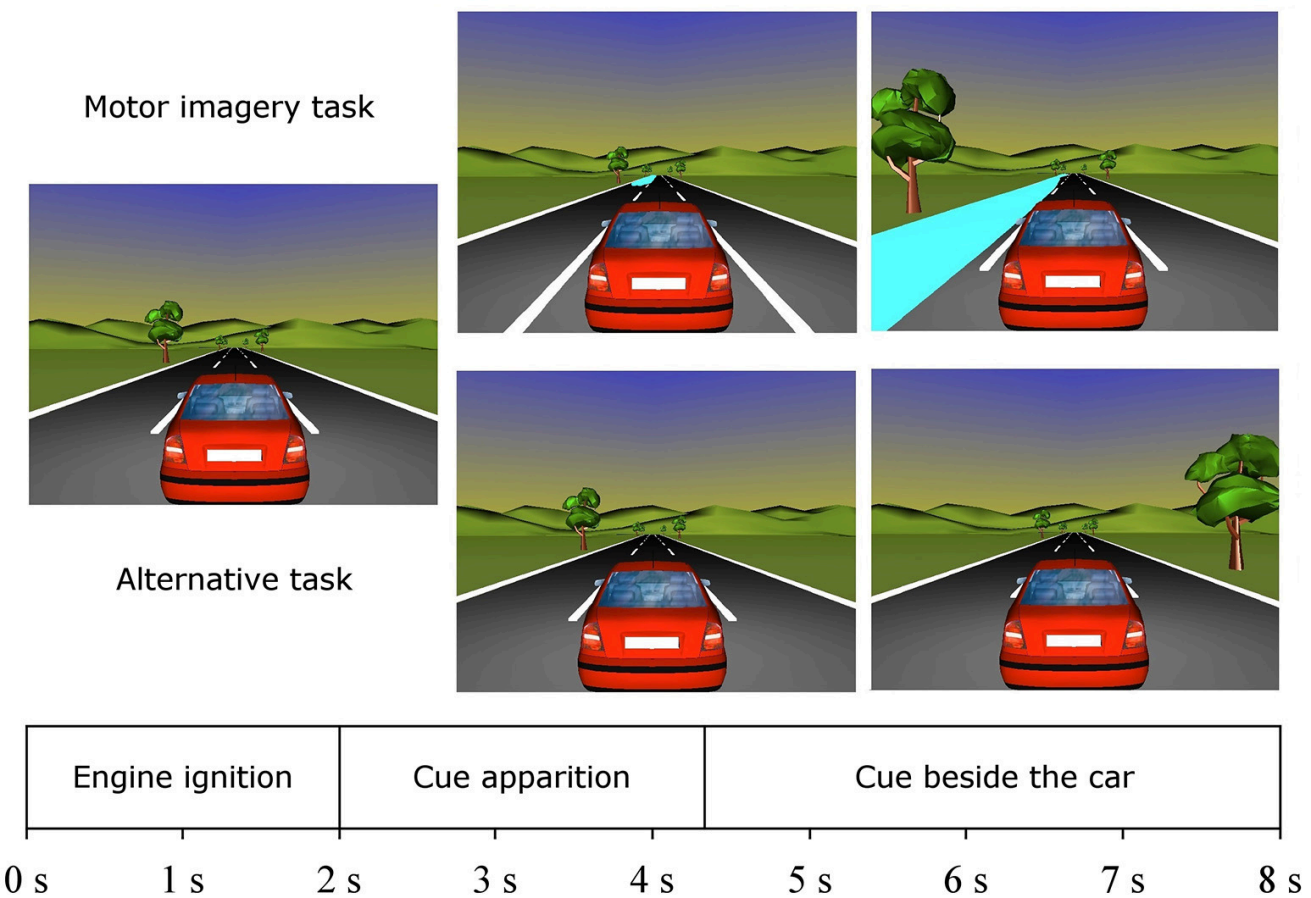
Timing of calibration trials. Right-hand MI **(Top)** and alternative tasks **(Bottom)**.

The timing of the calibration virtual session ran as detailed below (Figure 4). Initially, a car was placed in the middle of the road and its engine started at the beginning of the trial. Then, after 2 s, the car started to move, resulting in the possible appearance of a water puddle on the left side of the screen, located next to the car from the instant 4.25 s until the end of the trial. If the water puddle was presented, the participant had to perform the right-hand MI task from the time he/she starts to see the puddle until the sound of the car's engine ceases. Otherwise, if the puddle did not appear, the user should concentrate on performing the alternative task along the trial, i.e., in the time interval from 2 to 8 s of the trial. The calibration was divided into four blocks of 40 trials −20 of MI and 20 of the alternative task randomly ordered—to prevent fatigue and let the users rest between blocks. Also, there was a short random variable rest of 0.5–3 s between trials so as to be able to perform any movement that should not be performed during the trials. This phase lasted for approximately half an hour, excluding the time needed to set up the EEG recording equipment. Data from this phase were processed by the aforementioned algorithm to obtain the participant's reactive frequency band and optimal parameters of the LDA classifier. At this point, those participants whose EEG data could not be classified with an error rate lower than 30% were excluded. The virtual car environment was developed with VRML 2.0 and presented to users on a 15.6-inch laptop screen.

#### Navigation session with the brain-controlled wheelchair

**Figure 5:**
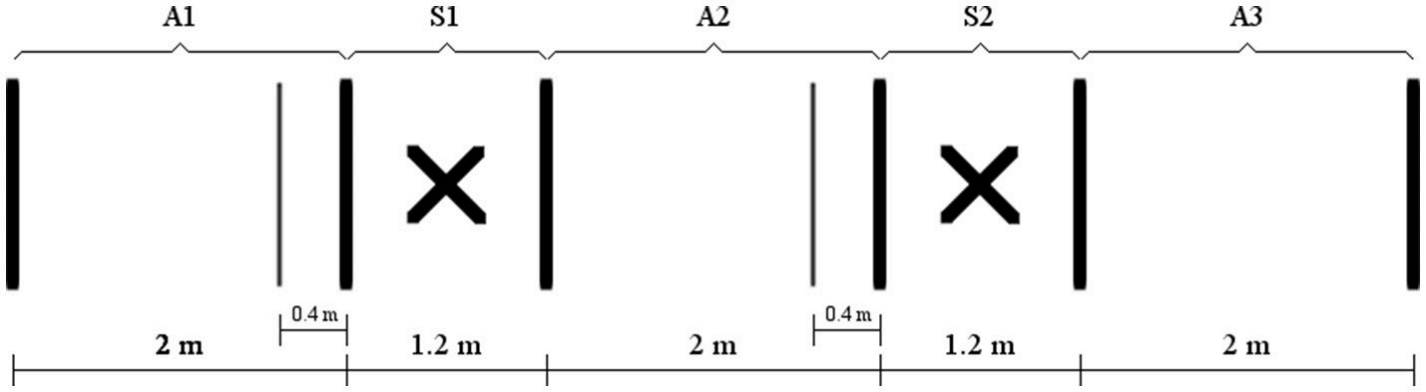
Path to complete in the navigation task. Advances sections from first to third were denoted as A1, A2, and A3, respectively, while the two stop sections were denoted as S1 and S2.

The path to complete consisted in an 8.4 m straight section in which the user had to get through three forward and two stop sections (Figure 5). The participants' objective was to complete the advance sections in the shortest time possible while in the stop sections the BCW should be stopped for up to 60 s, it not being necessary to perform this stop time in a single stop. Acoustic cues were used to inform the subject about sections changes and the time reached. Specifically, 40 cm before the stop zone, the word “arriving” was used and once inside it “inside” could be heard. Once the goal time (60 s) was reached, the user received the “timeout, continue” command, indicating the stop task had been successfully completed. If this time had not been completed when the user went out of this area, “out, continue” could be heard, indicating he/she was no longer in the stop zone and should now focus on the forward section. All indications were given in Spanish language, known by all participants.

The path should be completed at least twice by participants, one time in each control mode, i.e., continuous and switch modes. The order presentation was counterbalanced to prevent fatigue or a learning effect. The total time of the navigation session was around 45 min, including both the training and the testing. The training consisted in a first contact with the BCW, controlling it at free will and understanding how it worked in practice. Instead, the testing phase involved the completion of the path described above. Users were invited to carry out the test a second time after each control mode and before trying out the next one. In the cases where the user decided to complete the second run for the current control paradigm, only the one with the highest performance (using the *performance factor*, see section Evaluation) was included in the results. Therefore, the comparison was made with the runs with the best performance for each control mode.

In addition, a NASA-TLX questionnaire (Hart and Staveland, 1988) was completed after ending each navigation paradigm. In the same way, at the end of the session, two *ad hoc* tests were completed in order to know the users' opinions and experience during the navigation session.

### Evaluation

The evaluation was based on the definition of usability given by the International Organization for Standardization (1998), which considered three factors: effectiveness, efficiency and satisfaction.

#### Effectiveness

In order to analyse the performance controlling the BCW, we considered two basic parameters: (i) the number of command selections and (ii) measures related to the time employed in the advance and stop sections. From the number of command selections, we obtained statistical metrics based on the confusion matrix. Regarding the time measures, three *ad hoc* metrics were obtained that reflected the users' performance.

##### Confusion matrix metrics

Metrics related to the confusion matrix correspond to users' command selections (i.e., the bar exceeds the threshold for a longer time than a given “selection time”) depending on their intent and what actually happened (Mason et al., 2006). In this matrix, selections and non-selections are denoted as “positive” and “negative” respectively and the output as “true” or “false” depending on whether these selections were desired or not. This desired-output relation classified each selection as one of four possible categories in the matrix: *true positive* (*TP*)*, false positive* (*FP*)*, true negative* (*TN*), *and false negative* (*FN*). In order to make the comparison among subjects' performance easier, we considered 1 s time slots when analyzing the results; i.e., if a command was held for 4 s, this was considered as four command selections. It is worth remembering that in the case of the continuous mode, the forward command is “positive” and the stop command is “negative.” On the other hand, in the switch mode the “positive” selections are those changing the state of the BCW, so the first selection of a command is considered a “positive,” but keeping the same command active for several seconds is considered as “negative.”

The following metrics were used:

(i) *True positive rate* (*TPR*; Equation 1) indicates the user's ability to select the desired command.
  (1) $$\begin{array}{c} TPR=\frac{\sum TP}{\sum \left(TP+FN\right)} \end{array}$$
(ii) *True negative rate* (*TNR*; Equation 2) indicates the user's ability to avoid unwanted commands.
  (2) $$\begin{array}{c} TNR=\frac{\sum TN}{\sum \left(TN+FP\right)} \end{array}$$
(iii) *Positive predictive value* (*PPV*; Equation 3) indicates which of the user's selections are correct.
  (3) $$\begin{array}{c} PPV=\frac{\sum TP}{\sum \left(TP+FP\right)} \end{array}$$
(iv) *Negative predictive value* (*NPV*; Equation 4) indicates which of the user's non-selections are correct.
  (4) $$\begin{array}{c} NPV=\frac{\sum TN}{\sum \left(TN+FN\right)} \end{array}$$
(v) *Accuracy* (*ACC*; Equation 5) shows the level of overall performance.
  (5) $$\begin{array}{r} ACC=\frac{\sum TP+TN}{\sum \left(TP+TN+FP+FN\right)} \end{array}$$

An illustrative example of a classification sequence into the four possibilities of the confusion matrix (i.e., *TP, FP, TN*, and *FN*) is shown in Table 1. As said above, the classification was updated one time per second, so as the example has 10 s, there will be 10 different classifications. The objective of the table is to show how the classification will depend on the paradigm handled.

**Table 1:** Example of classification according to the confusion matrix.

**Time (s)**	**Command**		**Classification**	
	**Desired**	**Observed**	**Continuous**	**Switch**
0–1	Forward	Forward	TP	TP
1–2	Forward	Forward	TP	TN
2–3	Forward	Forward	TP	TN
3–4	Forward	Forward	TP	TN
4–5	Stop	Forward	FP	FN
5–6	Stop	Stop	TN	TP
6–7	Stop	Stop	TN	TN
7–8	Forward	Stop	FN	FN
8–9	Forward	Forward	TP	TP
9–10	Forward	Forward	TP	TN

*True positive (TP), false positive (FP), true negative (TN), and false negative (FN)*.

##### Time-related metrics

Besides the confusion matrix metrics, we considered that new metrics related to each specific command (forward and stop) could be appropriate in order to better evaluate the performance of each control mode in the different commands. These metrics are related with the time employed in the advance and stop sections in relation to the minimum and maximum time required in each section, respectively. Two ratios are defined:

(6) $$\begin{array}{r} Advance\;performance\;ratio\;\left(APR\right)=\frac{At_{\min}}{At_{o}} \end{array}$$

where *At*_*min*_ is the minimum time necessary to complete advance sections, 11 s, while *At*_*o*_ will be the observed time, i.e., the time executed by the user.

(7) $$\begin{array}{r} Stop\;performance\;ratio\;\left(SPR\right)=\frac{St_{o}}{St_{\max}} \end{array}$$

where *St*_*max*_ is the maximum time required to complete the stop section task, 60 s, and *St*_*o*_ is the observed time, i.e., the time executed by the user. If the user stayed in the stop section for 60 s, the time needed to leave it was not included in any metric, neither for the *SPR* nor *APR*.

These equations induce the idea that a good performance will show a lower time to complete the advance sections (never under 11 s, which is the minimum time necessary to complete 2 m by the wheelchair) and a longer stop time (never exceeding 60 s, the time subjects were asked to remain stopped). In this way, Equation 6 will show the user performance in the advance sections, while Equation 7 will do it in the stop sections. The results of both equations will range between 0 and 1, where 1 indicates the best performance.

Furthermore, to obtain a general measure of the users' performance, a factor considering these two ratios was defined:

(8) $$\begin{array}{r} Performance\;factor=APR\cdot SPR \end{array}$$

The fact of multiplying both ratios means that this factor presents a high value only in the case that both ratios are high as well. This means that a good performance is considered when both tasks can be voluntarily controlled. For example, in continuous mode, a system with an excellent performance in advances but deficient in stops (i.e., an uncontrollable BCW that always advanced) would have a high *APR*, but low *SPR*. If the mean value between these factors had been calculated, it would have offered a value near to 0.5; however, this performance would have been useless to allow users adequate control in a real environment. For this reason, as mentioned in section Navigation Session With the Brain-Controlled Wheelchair, the *performance factor* was used to select the best run with the BCW, since only one for each control mode was evaluated in the results section.

#### Efficiency

This factor has been mainly measured with the NASA-TLX questionnaire (Hart and Staveland, 1988), whose aim is to measure the user's workload executing a specific task once he/she has ended it. It is composed of six subscales (*mental demand, physical demand, temporal demand, performance, effort*, and *frustration*) in a scale ranging from 1 to 10 by users. Then, the participants have to indicate the relative contribution of the factors to their workload through 15 paired comparisons (e.g., *mental demand* vs. *physical demand*). A weighting average technique was used to compute the contribution of each subscale to the total workload. The total workload ranges between 0 and 100, while the weighted subscales are from 0 to 33.3. This questionnaire was applied two times, one for each control mode in the navigation task.

In addition, an *ad hoc* questionnaire about the experience controlling the wheelchair relative to relaxation, tiredness and performance (*ease to stop, ease to move forward, presence of false positives*, and *presence of false negatives*) was filled out by each participant at the end of the session. The variables of these questionnaires were ranged from 1 to 10 and written so that users could easily understand them (i.e., avoiding technical language).

#### Satisfaction

Satisfaction was measured employing another *ad hoc* questionnaire, whose items ranged from 1 to 10, to determine the comfort and subjective opinions of the user. These metrics were: *understanding of paradigm, control sense, motion smoothness, suitability*, and *efficacy of the paradigm*. In addition, at the end of the test users were asked to choose their favorite paradigm and to explain their choice.

## Results

This section will be divided into two parts in reference to the calibration and navigation tasks. Likewise, the navigation task part will be in sections for the usability factors mentioned above: effectiveness, efficiency and satisfaction. All the analysis performed was sample characteristics dependent, i.e., parametric or non-parametric, which means that mainly *t* Student and Wilcoxon tests were used for paired means comparison, respectively.

### Calibration session

The reactive band power features and minimum error rate obtained for each subject are presented in Table 2. On average, the minimum error rate was 23.70 ± 8.68%. Of the 15 subjects, five (P2, P9, P10, P13, and P15) had error rates above the cut-off point of 30% and did not continue with the study.

**Table 2:** Results of the calibration session.

**User**	**Frequency band (Hz)**	**Minimum error (%)**
P1	7–17	8.81
P2	13–16	31.31
P3	12–14	18.44
P4	12–17	17.94
P5	5–15	20.94
P6	11–15	22.38
P7	10–14	22.13
P8	10–16	23.06
P9	7–10	30.31
P10	11–16	35.19
P11	9–12	25.06
P12	7–12	15.81
P13	5–12	35.44
P14	10–14	11.63
P15	10–17	39.06
Mean	9.27 ± 2.52 to 15.47 ± 2.17	23.7 ± 8.68

### Navigation session

#### Effectiveness

In Table 3, the values of the different confusion matrix parameters obtained during the navigation task for each subject are shown. Regarding these measures, significant differences between continuous and switch mode were obtained for each of them: *TPR* [*t*_(9)_ = 3.583; *p* = 0.006], *PPV* [*t*_(9)_ = 11.983; *p* < 0.001], *TNR* [*Z* = 2.803; *p* = 0.005], *NPV* [*t*_(9)_ = −3.154; *p* = 0.012] and *ACC* [*t*_(9)_ = −2.517; *p* = 0.033].

**Table 3:** Results of the confusion matrix's parameters for each user and control mode.

**User**	**Classification matrix's measures**									
	**TPR**		**PPV**		**TNR**		**NPV**		**ACC**	
	**Continuous**	**Switch**	**Continuous**	**Switch**	**Continuous**	**Switch**	**Continuous**	**Switch**	**Continuous**	**Switch**
P1	0.55	0.32	0.79	0.54	0.84	0.94	0.63	0.86	0.69	0.83
P3	0.58	0.22	0.82	0.57	0.93	0.94	0.81	0.76	0.82	0.74
P4	0.28	0.17	0.84	0.60	0.92	0.94	0.45	0.70	0.53	0.69
P5	0.55	0.15	0.72	0.60	0.83	0.94	0.71	0.66	0.71	0.65
P6	0.47	0.27	0.82	0.64	0.89	0.94	0.62	0.76	0.68	0.74
P7	0.30	0.22	0.68	0.56	0.64	0.93	0.25	0.74	0.39	0.72
P8	0.72	0.15	0.71	0.56	0.67	0.94	0.68	0.70	0.69	0.69
P11	0.44	0.23	0.77	0.55	0.57	0.91	0.24	0.72	0.47	0.70
P12	0.19	0.35	0.77	0.53	0.73	0.86	0.16	0.74	0.28	0.70
P14	0.59	0.35	0.70	0.53	0.70	0.87	0.59	0.76	0.64	0.72
Mean	0.47 ± 0.17	0.24 ± 0.08	0.76 ± 0.06	0.57 ± 0.04	0.77 ± 0.13	0.92 ± 0.03	0.51 ± 0.22	0.74 ± 0.06	0.59 ± 0.17	0.72 ± 0.05
Satatistical test value	3.583		11.983		2.803		3.154		2.517	
*p*-value	0.006		0.001		0.005		0.012		0.033	

*True positive rate (TPR), positive predictive value (PPV), true negative rate (TNR), and negative predictive value (NPV). The two last row corresponds to the t_(9)_ Student comparison between continuous and switch modes for TPR, PPV, NPV, and ACC. In the case of TNR comparison, the Z-test value was calculated instead t*.

Table 4 shows the time spent by users executing each of the two BCW commands—move forward or idle state—as well as the number of move forward selections done in each section of the path. The users' average time to complete an advance section offered significant differences between both control modes: 42.2 ± 28.7 s and 15.47 ± 3.43 s for continuous and switch modes, respectively [*Z* = −2.803; *p* = 0.005]. Significant differences were obtained for stop sections too: 42.45 ± 16.01 s and 24.35 ± 10.94 s, for continuous and switch modes, respectively [*t*_(9)_ = 2.756; *p* = 0.022]. Regarding to the reaction time to stop the wheelchair when the user was advised that he/she was in the stop section, there was no significant differences between control modes: continuous (2.55 ± 1.5 s) and switch mode (3.55 ± 1.32 s).

**Table 4:** Results of the user performance: times and forward command selections.

**User**	**Control mode**	**Section of the path**														
		**Advance 1**			**Stop 1**			**Advance 2**			**Stop 2**			**Advance 3**		
		**A**	**B**	**C**	**A**	**B**	**C**	**A**	**B**	**C**	**A**	**B**	**C**	**A**	**B**	**C**
P1	Continuous	16	5	5	4	56	3	11	4	2	7	1	1	13	21	5
	Switch	11	0	1	7	26	1	10	2	1	8	43	2	13	5	2
P3	Continuous	13	4	2	6	54	2	11	0	0	2	58	1	16	17	5
	Switch	11	0	1	8	14	1	10	0	0	7	2	1	12	2	1
P4	Continuous	13	0	1	6	54	2	14	13	1	4	56	1	23	100	9
	Switch	13	8	2	7	19	1	7	7	1	7	53	2	15	13	2
P5	Continuous	16	6	4	12	48	8	16	18	5	7	53	3	18	11	5
	Switch	11	0	1	8	2	1	11	0	0	7	0	0	12	4	1
P6	Continuous	12	0	1	5	55	3	22	28	6	8	50	2	18	21	7
	Switch	11	0	1	8	26	1	11	4	1	8	6	2	11	2	1
P7	Continuous	14	48	3	12	10	4	22	64	7	13	34	4	18	17	4
	Switch	14	0	1	9	6	1	13	0	0	11	9	2	15	2	1
P8	Continuous	14	8	3	9	33	2	14	2	2	8	1	1	13	6	2
	Switch	11	0	1	6	36	1	12	17	2	7	0	0	11	2	1
P11	Continuous	19	23	5	9	20	2	13	2	3	6	0	0	18	38	5
	Switch	13	0	1	8	7	1	11	0	0	9	31	2	14	7	2
P12	Continuous	18	16	7	10	1	3	27	105	10	9	51	4	19	156	7
	Switch	14	3	2	7	24	2	12	2	1	8	4	1	13	5	3
P14	Continuous	14	1	2	14	39	7	18	16	4	9	15	2	22	21	8
	Switch	15	3	3	9	6	1	14	1	1	10	14	2	14	2	2

*While the letters “A” and “B” indicate the time (s) moving forward and keeping the position with the wheelchair, respectively; the letter “C” indicates the number of forward command selections. Thus, for example, the column relative to “Advance 2” and subcolumn “B,” would make reference to the time with the wheelchair stopped (B) in the second advance section*.

The average number of forward commands required to complete the advance sections was significantly different between conditions: 4.33 ± 1.58 s and 1.23 ± 0.52 s for the continuous and switch modes, respectively [*Z* = −2807; *p* = 0.005]. For the stop sections, similar results were obtained as the average number of forward commands was 2.75 ± 1.53 and 1.25 ± 0.42 for continuous and switch mode, respectively [*Z* = −2.439; *p* = 0.015].

**Figure 6:**
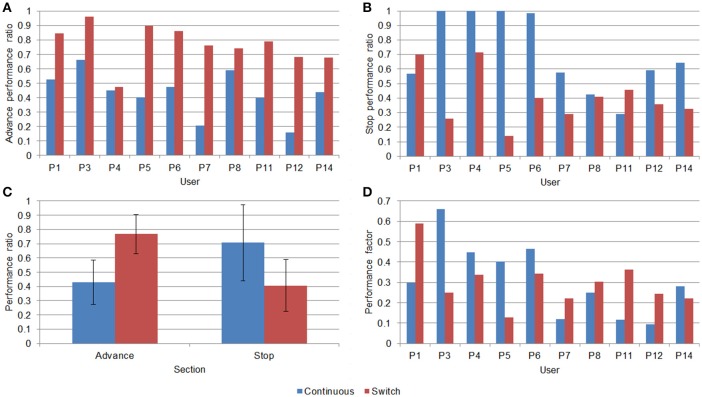
Results of the user performance: time-related metrics. **(A)** *Advance performance ratio* (*APR*) for each user. **(B)** *Stop performance ratio* (*SPR*) for each user. **(C)** Average values, with the corresponding standard deviation, for the APR and SPR. **(D)** *Performance factor* for each user.

Likewise, from the data in Table 4, performance ratios for each section and user can be calculated (Figure 6). A repeated measures ANOVA was performed to study the presence of main and interaction effects, involving the factors control mode (continuous or switch) and section type (advance or stop). The dependent variables included in this ANOVA were *APR* and *SPR*. The results showed significant differences in the interaction effect between the control mode and performance ratio variables [*F*_(1, 9)_ = 23.777; *p* = 0.001; $\eta_{p}^{2}$ = 0.725] (Figure 6C). These results showed that the control mode affects each of the variables differently, as we saw in the previous specific analysis relative to the time required to go over a section, offering a better performance ratio with the switch mode in advances [*t*_(9)_ = −6.363; *p* < 0.001] (Figure 6A) but better with continuous mode in stops [*t*_(9)_ = 2.756; *p* = 0.022] (Figure 6B). In addition, there are no significant differences between the *performance factor* related to the continuous mode (0.31 ± 0.18) and the switch mode (0.29 ± 0.12) (Figure 6D).

#### Efficiency

##### Workload

**Figure 7:**
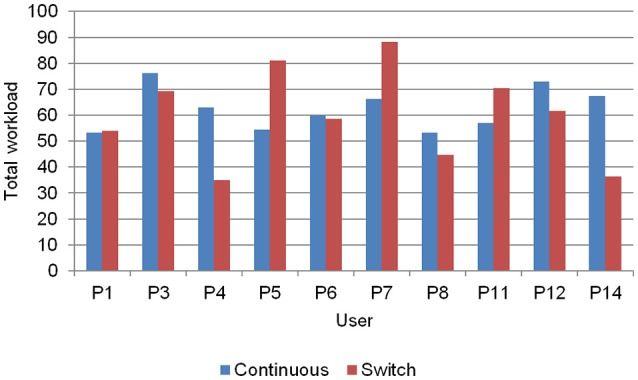
*Total workload* measured by NASA-TLX.

The average weighted factor results obtained with the NASA-TLX questionnaire are shown in Table 5, while the resulting *total workload* of each user is shown in Figure 7. No significant differences could be noticed between control modes.

**Table 5:** Average values and statistical result for the subjective measures reported by users.

**Subjective measures**	**Control**		**Wilcoxon test**	
	**Continuous**	**Switch**	***Z***	***p***
**NASA-TLX**				
Mental demand	22.03 ± 4.86	21.17 ± 6.95	−0.459	0.646
Physical demand	0.8 ± 2.2	0.17 ± 0.36	−0.816	0.414
Temporal demand	6.33 ± 4.01	7.53 ± 6.03	0.28	0.779
Performance	12.5 ± 5.24	8.87 ± 5.04	−1.955	0.051
Effort	14.97 ± 6.3	16.33 ± 8.49	0.561	0.575
Frustration	5.77 ± 7.06	5.87 ± 5.43	0.059	0.953
Total workload	62.4 ± 8.24	59.93 ± 17.95	−0.561	0.575
**SUBJECTIVE QUESTIONNAIRE FOR EFFICIENCY**				
Relaxed	8.29 ± 1.91	7.43 ± 2.44	−0.73	0.465
Tired	4.14 ± 2.03	4.86 ± 1.88	−0.73	0.465
Ease to stop	6.86 ± 2.17	5.43 ± 2.92	−1.084	0.279
Ease to move forward	5.57 ± 1.29	6.00 ± 1.85	−0.426	0.67
False positives presence	5.71 ± 1.67	6.29 ± 1.98	−0.687	0.492
False negatives presence	5.29 ± 1.75	5.43 ± 2.25	−0.69	0.49
**SUBJECTIVE QUESTIONNAIRE FOR SATISFACTION**				
Paradigm understanding	9.57 ± 0.49	9.14 ± 1.36	−1	0.317
Control sense	5.86 ± 1.73	4.86 ± 2.29	−0.681	0.496
Motion smoothness	5.43 ± 1.76	5.43 ± 1.76	−0.085	0.932
Suitability of the paradigm	7.29 ± 1.67	5.43 ± 2.44	−1.16	0.246
Efficacy of the paradigm	7.14 ± 1.73	6.86 ± 1.81	−0.632	0.527

##### Subjective questionnaire

The average answers given by participants at the end of the session in the usability questionnaire related to specific control mode features are shown in Table 5. Regarding these measures, only the scores of seven participants (P5, P6, P7, P8, P11, P12, and P14) are given as previous users were part of preliminary tests not using this questionnaire. No differences could be found in any factor of the two control modes with a Wilcoxon test.

#### Satisfaction

The different mean values obtained in another subjective questionnaire are shown in Table 5. As with efficiency, no significant differences were found between control modes in any factor of the subjective questionnaire (Wilcoxon test). In addition, 4 out of the 7 users who filled out the questionnaire preferred continuous mode vs. switch mode. A pattern can be observed in the explanation offered by three of the four users who preferred continuous mode (P5, P8 and P11; P7 did not explain their choice) according to the difficulty of changing mental tasks quickly and the requirement to maintain higher attentional levels in switch mode. Otherwise, two participants who preferred switch mode (P6 and P12; P14 did not explain his/her choice) declared that this mode implies a lower mental effort (P12) and that it was easier to control the BCW (P6).

## Discussion

This section will be divided into two subparts, one regarding the results obtained in this work, comparing the two control paradigms, and the other referring to previous works using the switch system or ERD/ERS signal based BCW.

### Discussion of the navigation control presented in this paper

First, the results obtained for the two control paradigms presented in the study will be discussed and compared in detail.

#### Effectiveness

According to the measure related to the general performance time, the *performance factor*, significant differences between paradigms are not observed but in specific sections of the path. On the one hand, the switch paradigm could be more effective in advance sections since it was possible to complete the same sections with fewer commands and better time-related metrics (i.e., time and *APR*). However, the opposite conclusion was obtained in the stop sections, where the continuous paradigm seems more convenient since users managed to stand still longer; however, the number of commands was significantly bigger too. The number of executed commands needed to leave the stop sections requires a more careful interpretation. Although the continuous mode was related with a larger number of forward commands in the stop section, when users selected a non-desired forward command in switch mode, they might not be able to stop the chair as quickly as needed, thus it made the BCW leave the stop section earlier than expected.

In general, these results may be explained by the false activations, i.e., *FPs*, which had a higher cost in switch mode than in continuous, in which the user could make these false selections with the slight cost of advancing just a few centimeters. Otherwise, in switch mode, these false activations could involve a larger displacement of the wheelchair, since quickly changing the movement state of the device could be difficult for some users (they should wait until the bar was lower than the threshold, then, they had to raise the bar again above the threshold during the “selection time” at least).

Measures related to the confusion matrix offered significant differences in all the considered variables. A pattern could be observed according to which the continuous mode obtained a better performance in the variables related to the *TPs* (*TPR* and *PPV*), while the switch mode obtained a better performance in the variables related to the *TNs* (*TNR* and *NPV*). These results make sense considering the desired results for each paradigm: the priority in the continuous mode was to have an adequate selection of the *TPs* in such a way that the displacement of the wheelchair was as fluid as possible. However, in the switch mode the intention was that the users could keep the state of the device as long as they wanted since, as we saw earlier, this could lead to a better performance. Nevertheless, in the most general measure of the confusion matrix, i.e., the *ACC*, the switch mode offered a better performance.

Despite these general differences in performance between control modes, it should be admitted that some users presented a better performance using one paradigm vs. the other. Thus, these results could support the idea that the paradigm should be chosen according the user preferences.

#### Efficiency

Regarding the usability questionnaires concerning efficiency, some points should be highlighted. At first, as expected, the most influential factor in the workload construct, measured with the NASA-TLX, was the *mental demand*, followed by *effort*, in both navigation paradigms. Most participants did not show appreciable differences in *total workload* between paradigms (Figure 7). However, for some participants one or the other paradigm noticeably involved more workload.

Regarding the subjective *ad hoc* questionnaire for efficiency, both paradigms shown similar values offering: (i) adequate results for relax state during the experiment, (ii) quite positive values for the metrics related to the ease to move or stop the wheelchair, although there were (iii) certain level of tiredness and (iv) quite negative values in reference at the presence of *FPs* and *FNs*.

#### Satisfaction

In reference to the subjective questionnaire for satisfaction: (i) all users adequately understood both paradigms, (ii) the control sense could be improved, especially for the switch mode, although there were no significant difference between them, (iii) both paradigms could be equally effective, (iv) the paradigm was not related to the motion smoothness as one might initially think, (v) the suitability of the paradigm offered acceptable scores, especially for the continuous mode despite there were no significant differences. The statements of those users who declared the continuous paradigm as their preference and explained their choice (P5, P8, and P11) agreed that the fast changes of mental tasks needed in the switch mode were difficult to perform.

### Discussion of previous works

Several BCI groups had studied a switch paradigm to control a BCW. The cases where the user can achieve an appropriate control of the alternative task present a certain parallelism with high level navigation paradigms (generally based on exogenous signal such as P300 or steady-state evoked potentials), since in these systems the user sends the order and he/she just has to wait while the command is executed. As it was shown in the introduction section, the brain switch control has been implemented in exogenous based BCWs with low level navigation to turn on/off the system, for example, in hybrid SSVEP based wheelchairs (Xu et al., 2012; Cao et al., 2014). The paper presented by Li Y. et al (2013) tested a hybrid (P300 and SSVEP) BCW where one the simultaneous detection of P300 and SSVEP stimulus was employed to change the advance state of the wheelchair (maintaining the advance or stop). Thus, this BCW applied the same concept used in the present paper with a MI based BCW. Furthermore, the SSVEP based BCWs are usually controlled through 4 or 5 control commands (Fernández-Rodríguez et al., 2016), so using the same stimulus to execute two allows that the number of commands can be incremented without the need of more stimuli.

Due to the specific experimental design to control the different devices and other factors such as the experience level of the participants, the comparison among switch systems will be limited to general aspects and to those which employed similar metrics. This problem has been declared in previous reviews about brain-controlled mobile devices (Bi et al., 2013; Fernández-Rodríguez et al., 2016) and BCI assessment (Thompson et al., 2013). As it is shown in Table 6, one of the main characteristics of the switch control presented here is the unbalance between the metrics related to the confusion matrix, especially between the *TPR* and *TNR*. This pattern was also obtained by Solis-Escalante et al. (2010). Regarding Müller-Putz et al. (2010), who calculated the *TPR* and the *PPV*, the presented switch proposal on the present paper shows more unbalanced *TPR* and *PPV* than them; however, the trend was the same: the *PPV* was higher than the *TPR*. Additionally, all switch control systems presented in Table 6 show the *TNR* as the highest measurement, and the *TPR* as the lowest. These results could be convenient since the switch control mode must have the ability to maintain the current state during the desired time. However, this capability of true negative detection and this low occurrence of false selections can lead to a system that is activated with difficulty. This could cause many false non-selections and thus result in a low value in the *TPR*. This imbalance can be a problem especially when the user needs to stop the BCW urgently, so in future proposals it would be necessary to include intelligent systems that assist navigation. However, as Ron-Angevin et al. (2017) concluded in their proposal on discrete control for the control of a BCW, the optimal values of these parameters depend on the type of system used, so they should be studied in future assessments.

**Table 6:** Results of the brain switch proposals: confusion matrix's measures.

**System**	**Control mode**	**TPR**	**PPV**	**TNR**	**NPV**
Present proposal	Continuous	0.47	0.76	0.77	0.51
	Switch	0.24	0.57	0.92	0.74
Müller-Putz et al., 2010	Switch	0.79	0.84	–	–
Solis-Escalante et al., 2010	Switch	0.46	–	0.86	–
Ron-Angevin et al., 2017	Discrete	0.79	0.77	0.85	0.81

*Brain-controlled wheelchair (BCW), event-related (des)synchronization (ERD/ERS), true positive rate (TPR), positive predictive value (PPV), true negative rate (TNR), and negative predictive value (NPV)*.

Regarding the workload, since there are no other studies using the NASA-TLX and controlling a BCW, it is difficult to discuss the observed values in the present study. In principle, it could only be compared with other studies that involve other tasks, such as the training to control the ERD/ERS signal (Felton et al., 2012), the handling of a complex P300 communication application (Riccio et al., 2011) or a simple P300 speller controlled by patients (Pasqualotto et al., 2015). Taking into account these previous works, whose *total workload* ranges between 30 and 67, approximately, it could be admitted that our values around 60 were adequate, especially if we keep in mind that the present work involve the control of a real wheelchair (i.e., the users move along with the BCW, they are not quietly seated in front of a computer).

## Conclusions

The performance shown by users during the navigation was heterogeneous, as were the workload and the evaluations through subjective questionnaires. Moreover, the results suggest that each control paradigm had specific advantages and drawbacks that must be taken into account. Specifically, a tendency was observed for the switch mode to enable a better performance than continuous mode in the advance sections, since the user could travel a longer distance with a single command selection. Otherwise, this advantage is converted into a drawback in the stop sections since in some cases users went through the stop section and could not stop the BCW. Thus, in these sections continuous mode offered better results. Another aspect to emphasize is the variability found in the *performance factor* between both controls for the same user, pointing to the possibility that what matters is not only the suitability of the paradigm, but also the preference and users' ERD/ERS modulations skills.

In short, this work has offered a detailed evaluation of two paradigms controlling a BCW considering the usability approach. To this end, many metrics were employed: those related to the objective performance of the user (such as time, number of selected commands, metrics of confusion matrix and even *ad hoc* measures such as the *APR, SPR* and performance factor), in addition to the subjective questionnaires, from the widely used NASA-TLX to specific *ad hoc* questionnaires.

For future works, it would be convenient to re-examine the control over different types of paradigms with trained users during several sessions, since it should be taken into account that all users (except user P4) were inexperienced at controlling these interfaces through their EEG signals. In addition, it may be interesting to study the application of new navigation paradigms that could have advantages over these two modes of control, so that users' performance and, therefore, their experience during the management would be as convenient and comfortable as possible.

## Author contributions

AF-R carried out the experimental sessions, collaborated in the experimental design, performed the statistical analysis and was a major contributor in writing the manuscript. FV-Á developed the BCW prototype, participated in the experimental sessions and was a major contributor in drafting the work. MB-S collaborated in the experimental design and carried out the experimental sessions. RR-A led the research, participated in the design of the BCW prototype, contributed in the experimental design and in the critical revision of the manuscript.

### Conflict of interest statement

The authors declare that the research was conducted in the absence of any commercial or financial relationships that could be construed as a potential conflict of interest.
